# Intraoperative Seizures During Awake Craniotomy for Brain Tumor Resection

**DOI:** 10.7759/cureus.43454

**Published:** 2023-08-14

**Authors:** Zara Shah, Saqib Kamran Bakhshi, Mujtaba Khalil, Faraz Shafiq, Syed Ather Enam, Muhammad Shahzad Shamim

**Affiliations:** 1 Research, Aga Khan University Hospital, Karachi, PAK; 2 Neurosurgery, Aga Khan University Hospital, Karachi, PAK; 3 Anaesthesiology, Aga Khan University Hospital, Karachi, PAK; 4 Surgery, Aga Khan University Hospital, Karachi, PAK

**Keywords:** epilepsy, glioma, brain tumor, awake craniotomy, intraoperative seizures

## Abstract

Background

Intra-operative seizures (IOS) can occur during awake craniotomies (AC) for brain tumors. They can potentially result in an increased risk of morbidity; however, literature is scarce on IOS, its risk factors, and predictors. This study aims to ascertain the frequency of IOS in patients undergoing AC and determine possible IOS predictors.

Methods

In this retrospective study, we reviewed the records of all patients who underwent AC for tumor resection at a single university hospital between January 2016 and December 2020. IOS was defined as any seizure, including partial or generalized, experienced by any patient at any time from the beginning of the procedure till the end of surgery.

Results

Two hundred patients underwent AC during the study period. Seven (3.5%) patients experienced IOS. Compared to the non-seizure group, no significant correlation existed with any demographic variable. No significant difference was seen between the initial complaints presented by the two groups. In addition, the post-operative course of the seizure group did not significantly differ from the non-seizure group. Due to the low frequency of IOS in our cohort, an extensive analysis to determine predictors could not be performed.

Conclusion

In this study, we observed a low frequency of IOS (3.5%) during AC. The possible predictors and risk factors must be further investigated in large cohorts; to help limit the consequences of this possible intraoperative complication.

## Introduction

Awake craniotomy (AC) is fast becoming the standard of care for the resection of brain tumors occurring in eloquent areas [1-5]. Up to 30% of patients undergoing AC can experience intraoperative seizures (IOS) [4]. Although IOS is one of the most common operative complications of AC, along with new neurological deficits, the predictors and consequences of this complication have not been studied in detail [5,6]. IOS can have an adverse impact on intra-operative monitoring, resulting in increased operative time, awake surgeries being converted to general anesthesia, more extended hospital stays, or, in the worst-case scenario, the inadequate extent of tumor resection and increased postoperative morbidity, worsening survival [7-10]. Yuan et al. reported the IOS rate at the beginning of surgery to be 11%, which increased to 35% towards the end of surgery [7].

Our rationale for this study was to ascertain the frequency of IOS in AC patients and to determine possible predictors of IOS. To achieve our objective, we retrospectively reviewed the data for all AC at our institute, specifically looking at patients who developed IOS during AC. To our knowledge, this is the most extensive study on this subject from a low- and middle-income country (LMIC).

## Materials and methods

This retrospective study, approved for an exemption by the Ethical Review Committee, consists of consecutive patients who underwent AC at Aga Khan University Hospital, Karachi, Pakistan, over a five-year period from January 2016 to December 2020. Data regarding the patient’s demographics, presenting neurological complaints, radiological findings, intra-operative complications, post-operative outcomes, tumor histology details, and functional status was reviewed and analyzed from patient medical records. Patients who had one or more episodes of seizures in the past, irrespective of the time interval between the seizure and tumor presentation, were considered to have a positive history of seizures. Additionally, the Karnofsky Performance Scale (KPS) was used to assess the functional status of the patients.

The seizure group was defined as patients who experienced any type of seizure, including partial or generalized (tonic-clonic seizures or loss of consciousness resulting in communication difficulties with the patient) at any time from the beginning of the procedure till the end of the tumor resection. The non-seizure group comprised the rest of the patients who underwent AC for tumor resection without experiencing any such event during the procedure. All patients underwent cortical and subcortical mapping with bipolar electrodes with incremental current use from 1mA to 4mA.

Anesthetic management of AC patients and management of IOS

All patients underwent a thorough preoperative anesthetic assessment, during which they were counseled and prepared for the procedure. The awake-throughout approach (AT) was adopted for the ACs, in which perioperative anesthesia was provided in the form of a scalp block through intermittent doses of either fentanyl or an infusion of dexmedetomidine for continuous conscious sedation. The depth of sedation was monitored using the Bispectral Index (BIS), which measures the brain's electrical activity on a scale of 0-100. All patients were managed prophylactically for seizures with preoperative levetiracetam as per our institution's protocol; any patients with unremitting seizures on levetiracetam are managed by the Neurology team, and second antiepileptic drugs are started. IOSs were initially managed by insufflating the surgical field with an ice-cold lactated ringers solution. Non-resolving seizures were then managed with incremental boluses of midazolam, which were then followed by a loading dose of either valproic acid or levetiracetam.

Statistical analysis

The sample size was determined by the number of awake craniotomy cases during the defined study period. Data was collected on a self-designed proforma containing details of demographics, surgery, and IOS. Analysis was done using IBM Corp. Released 2013. IBM SPSS Statistics for Windows, Version 22.0. Armonk, NY: IBM Corp. Categorical variables were reported as frequencies and percentages. Quantitative data were reported as mean (± SD). The normality of numerical variables was assessed using the Shapiro-Wilk test. Quantitative variables were compared using the independent t test/Mann-Whitney U tests for assessing the relationship of independent variables with outcomes, i.e., IOS vs. non-IOS group. Categorical variables were assessed by Chi square/Fisher exact tests. A p-value of < 0.05 was considered significant.

## Results

Of the 200 ACs performed during the study period, seven (3.5%) patients experienced intraoperative seizures. Six (85.7%) of the seven patients were males. The age of the patients ranged from 21 to 46 years old. Only one (14.2%) patient had a diagnosis of diabetes, while the other patients had no known co-morbid conditions. Six patients (85.7%) had presented with a history of seizures; three (42.9%) complained of headaches, and motor weakness was noted in three (42.9%) patients. Four patients (57.1%) had a preoperative KPS of 80. Pre-operative magnetic resonance imaging (MRI) revealed that most of the patients (five patients, 71.4%) had left sided lesions. Additionally, no patient from the seizure group had undergone a redo craniotomy, and neither had received radiation to the brain.

Intraoperative neurological deterioration was experienced by two (28.6%) patients, and two (28.6%) patients developed motor deterioration. During the post-operative hospital stay, one (14.2%) patient experienced seizures, and one (14.2%) patient developed headaches. There was no mortality in the seizure group. The demographics and clinical characteristics are summarized in Table 1.

**Table 1:** Demographics and clinical characteristics KPS: Karnofsky Performance Scale

Demographics and Clinical Characteristics (n = 7)	
Age years, mean ± SD	30.7 ± 7.8 years
Gender	
Male	6
Female	1
Handedness	
Right	5
Unknown	2
Length of stay in the hospital (Mean ± SD)	3.14 days ± 1.46 days
Presenting Complaints	
Headaches	3
History of Seizures	6
Motor Weakness	3
Location	
Frontal	3 (Left=2, Right=1)
Temporal	2 (Left=1, Right=1)
Frontoparietal	1 (Left=1)
Parietooccipital	1 (Left=1)
Histology	
Oligodendroglioma II	3
Oligodendroglioma III	1
Astrocytoma II	1
Glioblastoma IV	2
Nature of malignancy	
High grade	3
Low grade	4
Intraoperative Issues	
Neurological Deterioration	2
Motor Deterioration	2
Venous Bleeding	1
Emesis	1
Postoperative Issues	
Seizures	1
Headaches	1
Motor deterioration	0
Post-Operative KPS	
90	2
80	3
50	1
40	1

Univariate analysis revealed that there was no significant age difference between the seizure group and the non-seizure group (30.7 ± 7.8 vs. 39.6 ± 11.9, P=0.053). Although there was most males in both groups, the gender of the patients did not significantly correlate with seizure development during AC (P= 0.657). Similarly, the prevalence of the recorded presenting complaints, including headaches (P=0.772), a history of seizures (P=0.197), and motor weakness (P=0.148), in the seizure group was not significantly different from the non-seizure group.

The grade of the resected tumor did not reach statistical significance (P=0.622). A history of seizures and headaches was reported in both groups during the post-operative stay; however, the analysis did not show any statistical significance (P= 0.855, P=0.516, respectively). Additionally, analysis of the post-operative data did not show any significant difference in the length of the hospitalization period between the seizure and non-seizure groups (3.14 ± 1.46 vs. 3.15 ± 1.72, P= 0.991). Table 2 compares the patient demographics and clinical characteristics of the seizure and non-seizure groups.

**Table 2:** Patient demographics and clinical characteristics; non-seizure awake craniotomy vs. seizure awake craniotomy

Clinical Characteristics	Non-Seizure AC	Seizure AC	P-values
Numbers	193	7	
Age years, mean ± SD	39.6 ± 11.9	30.7 ± 7.8	0.053
Sex M:F	3.7:1	6:1	0.657
Presenting Complains			
Headaches	70	3	0.772
Seizures	119	6	0.197
Motor Weakness	39	3	0.148
Lesion Hemisphere			
Right	84	2	0.701
Left	109	5	
Nature of Malignancy			
High grade	101	3	0.622
Low grade	92	4	
Hospitalization Days, mean ± SD	3.15 ± 1.72	3.14 ± 1.46	0.991
Post-Operative Complications			
Seizures	17	1	0.855
Headaches	14	1	0.516

A review of existing literature reporting significantly associated risk factors/ predictors and outcomes of IOS during AC yielded four studies with study samples similar to ours (200 cases) or larger. The most reported risk factors/predictors are the history of seizures and tumor location. Nossek and colleagues are the only authors to report a significant association between IOS and post-operative outcomes, with IOS resulting in a significantly longer post-operative hospitalization stay [2]. Table 3 summarizes the reported predictors/risk factors of IOS during AC. 

**Table 3:** Studies reporting IOS during AC (200 cases or more) HIC: High-income country

Author, year	Number of Cases	IOS rate	Significantly Associated Predictors/Risk Factors	Reporting region
Current Study	200	3.5%	No significant association was found	LMIC
Lanthier, 2021 [4]	581	5%	Tumor location (frontal lobe), preoperative radiotherapy, preoperative use of antiepileptic drugs, intraoperative use of dexmedetomidine, intraoperative stimulation mapping	HIC
Blanshard, 2000 [11]	241	6.2%	-	HIC
Choi, 2019 [12]	416	24%	Alterations in genes coding for Recepter Tryosine Kinsase History of seizures and preoperative use of antiepileptics were negatively associated	HIC
Boetto, 2015 [13]	374	3.4%	No significant association was found	HIC
Nossek, 2013 [5]	477	12.6 %	History of seizures, tumor location (frontal lobe), young age	HIC
Sacko, 2011 [14]	214	5.7%	-	
Jumper, 2011 [15]	611	3%	History of seizures, tumor location (frontal lobe)	HIC
Kim, 2009 [16]	309	9%	History of seizures	HIC
Serletis, 2007 [17]	610	11.8%	-	HIC

## Discussion

Several authors have explored possible predictors of IOS during AC for brain tumor resection, including patient demographics, history of seizures, tumor location, and pre-operative antiepileptic use. To the best of our knowledge, we have reported the largest study from an LMIC reporting IOSs during ACs. In our study, seven (3.5%) of the 200 patients who underwent AC for brain tumor resection experienced IOS. None of the ACs were converted to general anesthesia due to AC failure, and there were no mortality cases. Compared to the non-seizure group, we found no statistically significant differences in the recorded patient demographics, the clinical presentation of the tumor, or the postoperative course.

The frequency of IOS in our patient cohort was 3.5%, which is similar to reports by Gupta et al. (3.8%) and Boetto et al. (3.4%) [13,18]. Notably, the incidence of IOSs during AC ranges from 3% to 30%, highlighting that the frequency of IOS in our cohort lies in the lower range of the observed IOS rates [4]. Although Nossek et al. reported that younger patients with brain tumors are at a higher risk for experiencing IOS during AC, other studies and our own data do not support this (p-value from our data=0.053) [4,5,13,19-21]. Tumor location has been strongly associated with IOS during AC. Frontal lobe involvement has been reported to be associated with a higher risk of IOS development [4,5,15], along with the Rolandic area and supplementary motor area (SMA) [9,20]. However, we did not find any significant association between tumor location and the incidence of IOS, much like other authors, including Boetto et al. and Lettieri et al. [13,21]. The relationship between the history of pre-operative seizures and the risk of experiencing IOS has previously been investigated. In our cohort, we did not find a significant correlation between the incidence of IOS and a history of preoperative seizures, similar to the previous few reports [13,19,21,22]. However, Nossek and colleagues found a significantly higher prevalence of a history of seizures in patients who experienced IOS, which is also in concordance with reports in existing literature [5,10,15,20]. Interestingly, we found no significant predictor for developing IOS during AC in our cohort as we explored patient demographics, preoperative clinical presentations, and tumor characteristics for possible significant risk factors.

In our patient cohort, IOS did not have a significant impact on postoperative outcomes during their hospital stay. The hospital stay was not significantly prolonged in the seizure group, which is contrary to the report by Nossek et al. The authors reported a significantly prolonged hospitalization period for the patients who experienced IOS compared to the non-seizure group [5]. Our study demonstrated that IOSs during awake surgery are most likely not to have an effect on the incidence of postoperative complications. Although both groups experienced post-operative headaches and seizures, the difference between the two groups did not reach statistical significance. Spena and colleagues reported similar findings, as the authors found no association between IOS and post-operative seizures [20]. Interestingly, there were no complaints of post-operative motor deterioration in our seizure group, which can be compared to the study by Nossek et al., in which post-operative short-term motor deterioration was reported in a higher percentage of patients who experienced IOS (20% vs. 10.1%, P = .02) [5]. Additionally, there were no cases of perioperative mortality, and none of the procedures had to be aborted or required conversion to general anesthesia. 

Our study is limited by a small sample size of AC patients and an even smaller seizure group, which precludes any meaningful derivation of possible predictors or risk factors. Although all the patients undergoing AC received a loading dose of an antiepileptic before the start of the procedure, we could not report the pre-operative use of antiepileptics due to inconsistency in patient records. Roca et al.’s review on the subject highlights the lack of studies assessing the relationship between IOS and AC [8]. Due to incomplete patient data, we could not study specific previously reported predictors for developing IOS, such as the isocitrate dehydrogenase 1 (IDH1) status of the tumor, preoperative tumor volume, and tumor margins. We, however, feel that our study will be a significant contribution to the existing literature, mainly since the existing literature on AC and IOS has limited representation from LMICs. Another important aspect warranting further research is the possible difference in the rates of IOS between patients who underwent awake throughout anesthesia technique or sleep-awake-sleep protocol.

## Conclusions

Our study shows that a relatively low percentage of patients can experience IOS during AC. This report aims to identify variables associated with the occurrence of IOS to help minimize the incidence of IOS. However, future studies with large study samples, specifically from LMICs, are needed to assess IOS's predictors and risk factors during AC.
